# Helicopter Emergency Medical Service in Fars Province: The Referral Trauma Center of South of Iran

**Published:** 2012-05-30

**Authors:** M J Moradian, B Rastegarfar, R Salahi, H R Abbasi, Sh Paydar, M R Rastegar, M Dehghani, S Mousavi, E Shirzad, M Khorrami, M Esnaashar, Sh Bolandparvaz

**Affiliations:** 1 Fars EMS, Shiraz University of Medical Sciences, Shiraz, Iran; 2 Department of Disaster Public Health, School of Public Health, Tehran University of Medical Sciences, Tehran, Iran; 3 Department of Disaster and Emergency Health, National Institute of Health Research, Tehran University of Medical Sciences, Tehran, Iran; 4 Trauma Research Center, Shiraz University of Medical Sciences, Shiraz, Iran; 5 Education Development Center and Student Research Committee, Shiraz University of Medical Sciences, Shiraz, Iran

**Keywords:** Iran, Helicopter, Ambulance, Air transfer, Trauma

## Abstract

**Background:**

Considering the limited available resources, high cost of the helicopter emergency medical service (HEMS), and high load of trauma patients especially in our centers, a careful assessment of HEMS in our center seemed to be necessary for trauma patients.

**Methods:**

From April 2001 to September 2007, the data of all patients transferred by HEMS were extracted including: Annual number of services, clinical category, number of proper or improper services, and rescue time for HEMS and ground ambulance. The criteria for the properly transferred group included: Death or being operated in the first 24 hours of admission, admission in ICU care units, and transfer of more than three patients in one mission. Others were considered as improper group.

**Results:**

In this period through 185 flights, 225 victims were transferred. The most common reason of HEMS dispatching was trauma. The most difference of rescue time between ground ambulance and HEMS was recorded in Lamerd that was transferring patients with HEMS needed 3 hours less than ground ambulance. However, in Sarvestan, Dashte-Arjan, and Marvdasht, transferred patients with ground ambulance needed less time than air transfer. Most of transferred patients were from Kazeroon, Nourabad and Lamerd respectively while 46.3% of patients were in the proper group, and the rest were considered as improper group.

**Conclusion:**

Our study revealed that helicopter dispatch to the cities like Lamerd, Lar, Khonj, Abadeh can be more effective, whereas, for the towns like Marvdasht, Dashte-Arjan, Sarvestan, Sepidan, Saadatshar, Tang Abolhayat use of HEMS should be limited to specific conditions. Our study showed inclusion of physicians in the decision making team increased the number of transferred cases.

## Introduction

The necessity of the evacuation of casualties from the battlefield and taking care of them led to the development of ambulances to transport the injured patients. This idea was experienced in Burma during World War II.[1][2] Formal use of helicopter emergency medical service (HEMS) was experienced in the Korean and Vietnam wars.[1][3] These successful experiences brought the idea of integrating HEMS into the modern health systems to help to transfer patients more quickly.[1]

Fars Province is located in the south of Iran with an area of 122400 square kilometers and includes 24 towns. In addition, it is considered as the supporting province for adjacent provinces by the Ministry of Health, Treatment, and Medical Education. Therefore, Fars Aviation Emergency Medical Service Center was founded in 2001 by one "205 helicopter" at first and then by one "MIL-17" helicopter instead since 2004. Considering the limited available resources, and high cost of the HEMS, high load of trauma patients especially in developing trauma centers, a careful assessment of HEMS in our center seemed to be necessary to provide a better service for trauma patients.

## Materials and Methods

**Fig. 1:**
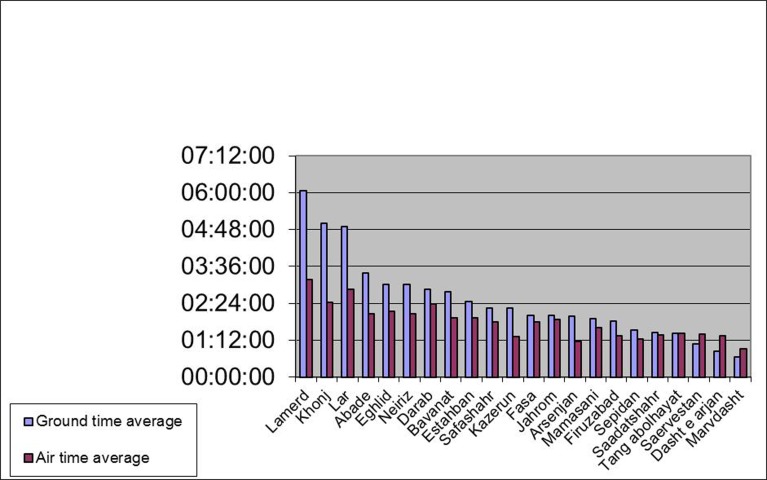
Comparison between ground and air transfer times in different towns in Fars Province.

In a retrospective study, the data related to all the injured patients transferred by HEMS of Fars from April 2001 to September 2007 were extracted. The data included: Annual number of services, clinical category, follow-up of the patients, and number of proper or improper services. Given the high costs of HEMS in comparison with ground transfer (more than 40 times higher per hour), we also calculated rescue time for HEMS and ground ambulance from different cities of Fars Province (Figure 1). Rescue time was defined as the duration between the time of sending the ambulance and the time of arrival of patients to the airport. The ground and air transfer times were extracted from the Ground Mission Transfer Form and the Air Rescue Data Form respectively (Figure 1).

To evaluate the effectiveness of HEMS in Fars Province, we divided the transferred patients into two category "Proper and improper groups" based on the following criteria. The criteria for the properly transferred group included: (i). Death or being operated in the first 24 hours of admission in hospital, (ii). Admission in ICU care units, (iii). Transfer of more than three patients in one mission. If the service was not consistent with the criteria for proper group, it was defined as improper group. To collect, organize and analyze the data from the mentioned forms, Access and Excel software were used.

## Results

From April 2001 to September 2007 through 185 flights, 225 victims with an average age of 26.5 years were transferred to equipped treatment centers of Shiraz, Southern Iran. As car accidents (75%), fractures (2.5%), falling down (2%), shut gun (1.5%), stab wounds (2%) were included in the trauma section, the most common reason of HEMS dispatching was related to trauma patients (83%), followed by internal medical events (7%), general surgery (4%), gynecology (4%) and pediatrics (2%) respectively. Our study showed that in 2005, after including one more physician in the decision making team, the number of transferred cases increased more than doubled compared to 2004.

As is indicated in Figure 1, the time needed by ground ambulance or helicopter ambulance to transfer patients from different cities to Shiraz airport (rescue time) varied among different cities. Figure 1 showed that the most difference of rescue time between ground ambulance and HEMS was recorded in Lamerd , that is, transferring patients with HEMS needed 3 hours less than ground ambulance; Tang-Abul-Hayat was the only area where ground and air transfer time was the same. However, in Sarvestan, Dashte-Arjan, and Marvdasht, transferring patients with ground ambulance required less time than air transfer.

According to Table 1, the cities with the most transferred casualties by HEMS were Kazeroon, Nourabad and Lamerd respectively (14%, 13% and 10% respectively). The phone calls with patients transferred by HEMS showed that 48.6% were currently healthy, 40% were died in hospital, 8.6% suffered from disabilities and one person is still in coma.

**Table 1:** Number of transferred casualties from April 2001 to September 2007.

**City**	**2001**	**2002**	**2003**	**2004**	**2005**	**2006**	**2007**	**Total**
Kazerun	-	3	2	2	7	10	2	26
Noor abad	1	1	5	5	7	5	-	24
Lamerd	1	2	1	-	9	6	1	19
Darab	1	2	1	1	2	6	1	14
Khonj	1	-	2	-	3	6	2	14
Fasa	-	1	2	-	2	2	-	7
Gerash	-	1	1	2	1	2	-	7
Firuzabad	-	2	1	-	-	1	2	6
Maharlu	1	-	2	2	-	-	-	6
Abaadeh	-	-	1	-	1	3	-	5
Lar	1	2	-	1	-	-	-	4
Marvdasht	-	1	2	-	1	-	-	4
Sarvestan	-	-	3	1	-	-	-	4
Bavanat	-	-	-	-	3	1	-	4
Abolhayat	-	-	1	1	1	-	-	3
Dashte-arjan	-	-	2	-	1	-	-	3
Eghlid	-	-	-	-	-	-	3	3
Khorambid	-	-	-	2	1	-	-	3
Arsanjan	-	-	-	-	2	-	-	2
Sepidan	-	1	-	-	-	1	-	2
Zarghan	2	-	-	-	-	-	-	2
Saadat-Shahr	-	1	-	-	-	-	-	1
Polekhan	-	1	-	-	-	-	-	1
Sivande Jadid	-	-	-	1	-	-	-	1
Gardane Ghader Abad	-	-	-	1	-	-	-	1
Estahban	-	-	-	1	-	-	-	1
Fasa Entrance	-	-	-	1	-	-	-	1
Gardanea Shul	-	-	-	-	1	-	-	1
Ashkanan	-	-	-	-	-	-	1	1
Beyza	-	-	-	-	1	-	-	1
Neyriz	-	-	-	-	1	-	-	1
Evaz	-	-	-	-	1	-	-	1
yasuj	-	-	-	-	1	1	-	2
Sirjan	-	-	-	-	1	-	-	1
Gardane Bajgah	-	-	-	-	1	-	-	1
Ghalat mountains	-	-	-	-	-	1	-	1
Bushehr	-	-	-	-	-	1	1	2
Kuhe Derak	-	-	-	-	-	1	-	1
Golestan	-	-	-	-	-	1	-	1
Asalooye	-	-	-	-	-	1	-	1
Jahrom	-	-	-	-	-	-	1	1
Meydane Tarebar	-	1	-	-	-	-	-	1
Bid-Zard	-	-	1	-	-	-	-	1
Total	9	19	26	21	48	48	14	185

The classification of air rescue flight according to the considered criteria reflected that 46.3% of the transferred casualties belonged to the proper group, and 53.7% of patients were considered as improper group.

## Discussion

Despite three decades of experiences with airtransferring multiple trauma patients, the effectiveness of HEMS has been remained controversial.[4] In a systematic review, Taylor et al.,[1] evaluated the cost effectiveness of HEMS. They evaluated eight studies which reported variant cost-effectiveness ratios including $3292 and $2227 per life year saved, $7138 and $12,022 per quality adjusted life year.[1] However, five studies had opposite viwes.[1]

In our study, patients were younger than patients transferred by HEMS in developed centers (26.5 years vs 40 years).[5] Therefore, by improving our emergency services and number of available helicopters, we will save more lifelong than developed countries because of having young population and higher load of trauma patients in our center.[6]

There is no doubt that HEMS is very useful in specific places with geographic difficulties[7] because it needs less rescue time than ground ambulance.[4][8] Comparing rescue time of HEMS and ground ambulance, we found that helicopter dispatch to the cities including Lamerd, Lar, Khonj, Abadeh can be more effective because rescue time of HEMS was less than ground ambulance; whereas helicopter dispatch to the cities including Marvdasht, Dashte-Arjan, Sarvestan, Sepidan, Saadatshar, Tang Abolhayat can be effective only in specific conditions such as conditions when it is difficult for ground ambulances to move because of road blockage or conditions in which the ground ambulances were not enough to transport high load of injured patients.

Our study showed that adding a physician to the decision making team in 2005,resulted in increasing the number of transferred cases to more than two times in comparison with 2004 and hence availability to advanced treatment improved as Lossius et al. stated that use of skilled and experienced personnel had important role to have effective HEMS.

Evaluation of effectiveness of HEMS can be performed upon predefined criteria for proper transfer. However, there is not a common criteria to dispatch helicopters among different trauma centers.[8] Overtriage is necessary to decrease the rate of undertriage, which is associated with high degree of mortality rate. Petrie et al.,[10] used following criteria to dispatch HEMS: Requiring admission to a critical care unit, death during transportation or in first 24 hours, or in the case of trauma, an injury severity scale score > or =12. Overall, 86.9% of missions were appropriate according to their definition, and overtriage rate was 13.1%.[10] Wigman et al.[4] reported that HEMS in Europe was at risk of increased rate of overtriage because of using mechanism of injury and patient characteristics to dispatch HEMS. An over-triage rate of 50% was acceptable by the American College of Surgeons to reduce the under-triage rate to 10%.[11]

Based on the predetermined criteria in our study, 46.3% and 53.7% of the rescue flights were proper and improper respectively. The reason of difference of over-triage in our study with previous studies might be due to the indicator of "less than three days of hospitalization" for improper transfers in our study. It can wrongly reflect a higher percentage of overtriaged or improperly transferred patients. The other reason is that in our study, trauma patients were the leading category of HEMS and trauma situations were associated with higher rates of over-triage than other situations because decision making in early undifferentiated trauma situations was difficult.[10] Moreover, there was not a flight at night and it was a limitation in our study.

Braithwaite et al.[12] reported that use of Injury Severity Score (ISS) between 15 and 60 was associated with decreasing mortality rate. Whereas, some authors believed that a combination of set criteria and manual selection by an experienced clinician might be the best choice to improve the sensitivity and specificity of HEMS.[8][13] However, considering high load of trauma in our center,[6] limited accessible helicopters, high expense of HEMS, and having different geographic characteristics from developed countries, it seems that an adjusted type of guidelines should be prepared for developing centers like our center.[14] Given the after-mentioned points, it seems crucial to reconsider the present indices and protocols for the purpose of decreasing expenses and increasing the effectiveness of HEMS.
